# Relationship between childhood trauma, parental bonding, and defensive styles and psychiatric symptoms in adult life

**DOI:** 10.47626/2237-6089-2020-0086

**Published:** 2021-10-22

**Authors:** Vitória Waikamp, Fernanda Barcellos Serralta, Luis Francisco Ramos-Lima, Cleonice Zatti, Lucia Helena Machado Freitas

**Affiliations:** 1 Programa de Pós-Graduação em Psiquiatria e Ciências do Comportamento Universidade Federal do Rio Grande do Sul Porto Alegre RS Brazil Programa de Pós-Graduação em Psiquiatria e Ciências do Comportamento, Universidade Federal do Rio Grande do Sul (UFRGS), Porto Alegre, RS, Brazil.; 2 Programa de Pós-Graduação em Psicologia Universidade do Vale do Rio dos Sinos São Leopoldo RS Brazil Programa de Pós-Graduação em Psicologia, Universidade do Vale do Rio dos Sinos (UNISINOS), São Leopoldo, RS, Brazil.

**Keywords:** Childhood trauma, defense mechanisms, parental bonding, psychopathology

## Abstract

**Introduction:**

A relationship between different types of childhood trauma, parental care, and defensive styles and development of psychiatric symptoms in adulthood is proposed in this study. Understanding the nature of this association is essential to assist psychotherapists who treat patients with a history of past trauma. This study aims to examine the associations between childhood trauma, parental bonding, and defensive styles and current symptoms in adult patients who sought care at an analytical psychotherapy clinic.

**Methods:**

The sample comprised 197 patients from an analytically oriented psychotherapy clinic. Participants responded to four self-report instruments that assessed, respectively, presence and frequency of several types of early trauma, type of parental attachment, styles of defenses, and current symptoms encompassing a wide variety of psychopathological syndromes.

**Results:**

Only 5% of patients reported not having experienced any traumatic experience in childhood. Several traumas such as emotional and physical abuse, emotional neglect, and physical neglect showed positive and significant associations with several dimensions of current symptoms, and also with parental bonding and defensive styles. When analyzed together with the other variables, defensive styles explained the level of psychological suffering caused by the symptoms.

**Conclusions:**

This study offers additional support for understanding the associations between childhood trauma, parental bonding styles, and defense styles and the psychiatric symptoms of patients in analytically oriented psychotherapy.

## Introduction

According to the World Health Organization,^1^ child maltreatment includes various forms of neglect and abuse that cause potential damage to health, development, and the child’s dignity. According to Bins et al.,^2^ there are four types of child abuse: physical abuse (use of physical force with the aim of hurting), sexual abuse (sexually stimulate, to obtain sexual satisfaction), emotional abuse (defined as using words and actions that shame, censor, humiliate, and permanently pressure the child), and neglect (depriving the child of something it needs, when this is essential to its healthy development). A single meta-analysis found to date has estimated that more than three quarters of children on the planet have had some moderate or severe experience of physical, sexual, and/or emotional abuse during 2015, affecting almost 1.5 billion children aged between two and 17.^3^

In Brazil, according to the Ministry of Human Rights,^4^ the main complaints of abuse of children and adolescents are negligence (73.07%), psychological abuse (47.07%), and sexual violence (24.19%). A single child or adolescent may be the victim of multiple types of harmful treatment, which is why the percentages exceed 100%. The data shows a jump from 76,171 records in 2016 to 84,049 in 2017. On average, 230 calls are made to the Ministry of Human Rights whistleblowing channel per day.

From a psychoanalytic perspective, psychic trauma means a “violent shock” capable of breaking the protective barriers of the ego, leading to lasting disturbances in the individual’s mind.^5^ If treated inappropriately, the severity and frequency of these traumatic experiences in childhood can predispose to manifestation of several psychiatric symptoms, especially anxiety, depression, and psychosis,^6-8^ as well as contributing to changes in the architecture and function of the brain in adulthood.^9,10^

Garland^11^ points out that the possibility of the individual recovering is associated with the quality of their relationships and primary care. This care is crucial for the structure of their psychic apparatus and for acquisition of important skills such as emotional regulation, reflexive function, and the ability to mentalize.^12^

Failures in primary relationships and exposure of individuals to childhood adversities can alter the course of normal development, leading to precarious psychic resources. This results in impairment of the symbolic capacity to represent traumatic experiences, leading the individual to become more susceptible to psychological suffering. It is therefore understood that a sufficiently healthy relationship between the child and their parental figures acts as a protective factor against development of psychiatric symptoms.^13,14^

Research has shown associations between childhood trauma, parental bonding, and psychiatric symptoms in adulthood. Catalan et al.^15^ investigated the relationships between different types of parental care, childhood trauma, and psychotic symptoms in adulthood in patients with borderline personality disorder, patients with a first psychotic episode, and healthy controls. They found positive associations between psychotic symptoms and the existence of childhood trauma in all groups. In addition, “affectionless control” was directly associated with the existence of trauma. From this same perspective, Marshall et al.^16^ explored these relationships and found that maternal “affectionless control” was significantly associated with depressive symptoms in adults.

In favor of protecting the ego in the presence of symptoms, in *The neuro-psychoses of defense*,^17^ Freud describes how defense mechanisms are unconscious psychological processes that aim to protect the individual from the internal perception of painful affective states. These mechanisms are characterized as a psychic phenomenon that appears in early childhood and are mainly influenced by attachment style.^18-20^ As stated by Gabbard,^21^ immature defenses allow individuals to maintain an illusion of emotional control when they experience a situation of helplessness.

According to Colovic et al.,^22^ anxiety and depression can be distinguished using certain defense mechanisms. Immature defenses were significantly more associated with depressed patients than with anxious ones. Their results did not confirm a significant difference in use of neurotic defenses between patients with anxiety and depressive disorders.

The social and occupational impacts of these adverse conditions reinforce the importance of considering the issue as a public health problem at all levels of prevention, especially in programs that promote learning of positive parenting and care skills.^1,23,24^

This study aims to examine the associations between child trauma, parental bonding, defensive styles, and current symptoms in adult patients who sought care at an analytical psychotherapy clinic. It is essential to study this relationship because child abuse is still being reported as a widespread problem worldwide and it has devastating impacts on mental health.

## Materials and methods

### Design

The present study has a quantitative, cross-sectional design.^25^ It is derived from a larger project coordinated by author Fernanda Serralta at LAEPSI (Laboratório de Estudos em Psicoterapia e Psicopatologia) at Universidade do Vale do Rio dos Sinos (UNISINOS), which aims to examine the associations between traumatic experiences, attachment style in childhood, and dysfunctions of personality in adult life and determine the impact of these variables on the processes and results of psychoanalytic psychotherapy. Data collection was carried out at an analytical psychotherapy outpatient clinic in Porto Alegre, Rio Grande do Sul, Brazil, and a database of 210 patients was generated.

### Sample

All patients over 18 years of age who sought care between April 2015 and October 2016 were included in the present study. The total sample had a mean age of 32 years (standard deviation [SD] = ±12.21) and a majority were female (69%). In terms of educational level, the sample was distributed as follows: elementary education (1.1%); high school started but not completed (6.3%); high school completed (16.4%); higher education (74%); and technical course (2.1%).

### Instruments

#### Childhood Trauma Questionnaire (CTQ)

The CTQ originally comprised 70 questions and was later condensed to produce a short version comprising 28 questions. This version was translated and validated for Portuguese by Grassi-Oliveira et al.^26^ and has the same properties as the original scale, investigating five dimensions of trauma: physical abuse (PA), emotional abuse (EA), sexual abuse (SA), physical neglect (PN), and emotional neglect (EN). Each dimension consists of five questions responded on a five-point Likert scale ranging from 1 never to 5 almost always. The remaining three questions comprise a scale used to control the reliability of answers. Bernstein & Fink^27^ achieved good indicators for the internal consistency of all of the subscales, calculating Cronbach’s alphas with medians varying from a = 0.66, for the physical neglect subscale, to a = 0.92 for the sexual abuse subscale. In a study with adult patients, the reliability coefficient was 0.87. For the present study, reliability was assessed by analyzing internal consistency using Cronbach’s alpha coefficients and McDonald’s omega, ranging from α = 0.7 and ω = 0.74 for physical neglect to α = 0.94 and ω = 0.95 for sexual abuse.

#### Defensive Style Questionnaire (DSQ-40)

The DSQ-40 was developed by Bond et al.^28^ to evaluate conscious derivatives of defense mechanisms. Andrews et al.^29^ validated and reorganized the instrument into its current form. The DSQ-40 aims to assess defense styles and is made up of 40 items associated with the defenses featured in the DSM-III-R. Each item is scored from 1 strongly disagree to 9 strongly agree. The instrument assesses 20 types of defenses with two items for each type. Defenses are divided into three factors: mature, neurotic, and immature. Four defenses are related to the mature factor (sublimation, humor, anticipation, and suppression); four are related to the neurotic factor (undoing, pseudo-altruism, idealization, and reaction formation) and twelve are related to the immature factor (projection, passive aggression, acting out, isolation, devaluation, ‘autistic fantasy’, denial, displacement, dissociation, splitting, rationalization, and somatization). The version adapted for Brazil was proposed by Blaya^30^ and has demonstrated reliability. It was evaluated with Cronbach’s alpha coefficients of 0.68 for the mature style, 0.71 for the neurotic style, and 0.77 for the immature style. Test-retest reliability analysis determined coefficients of 0.68 for the mature style, 0.71 for the neurotic style, and 0.81 for the immature style. For the present study, reliability was estimated in terms of internal consistency using Cronbach’s alpha and McDonald’s omega coefficients with results of α = 0.54; ω = 0.68 for the mature style, α = 0.82; ω = 0.85 for the immature style, and α = 0.58; ω = 0.72 for the neurotic style.

#### Brief Symptom Inventory (BSI)

The BSI is an abbreviation of the SCL-90 (Symptom Checklist – 90), an instrument widely used in several countries to assess symptoms of mental disorders and psychological distress.^31^ The instrument comprises 53 items scored on a 5-point Likert scale and assesses nine symptomatic dimensions (anxiety, phobic anxiety, depression, hostility, paranoid ideation, obsession-compulsion, psychoticism, interpersonal sensitivity, and somatization), producing the General Severity Index (GSI), the total number of positive symptoms, and the positive symptom index. The GSI is the most reliable indicator and the one most often used, being considered a general measure of psychological distress or suffering derived from the symptoms. The Brazilian Portuguese version was adapted by the team at the LAEPSI at UNISINOS from the Portuguese version by Canavarro.^32^ For this study, reliability was estimated using Cronbach’s alpha and McDonald’s omega, showing good internal consistency: α = 0.97 and ω = 0.97.

#### Parental Bonding Inventory (PBI)

The PBI comprises 25 questions about the subject’s father and mother scored from 0 to 3 on a Likert-type scale, each of which asks how similar a specific behavior is to the subject’s parents’ behavior up to the age of 16 years. The PBI measures two constructs: care and control. High scores on the care subscale indicate perceptions of affection and closeness, while higher control scores suggest excessive protection, surveillance, and infantilization. For mothers, the cut-off point for care is 27.0 and the cut-off point for control is 13.5; for fathers these cut-offs are 24.0 and 12.5, respectively. The Brazilian Portuguese version was prepared by Hauck et al.^33^ According to these authors, the various studies carried out with the instrument attest that it is a psychometrically robust measure, stable over time, and whose construct remains valid in the various different versions for other languages already developed and validated. Cronbach’s alpha and McDonald’s omega estimators were used in this study to assess reliability, with values for maternal and paternal care of α = 0.92 and ω = 0.93 and α = 0.91 and ω = 0.93, respectively. Maternal and paternal control reliability values were α = 0.85 and ω = 0.91 and α = 0.85 and ω = 0.90. Hauck et al.^33^ attested to the conceptual, semantic, functional, and operational equivalence of the instrument.

## Data collection procedure

Data collection took place in the context of the larger research project to which this study is linked. During screening conducted by a research fellow, the research was explained and subjects were invited to participate voluntarily. After signing the informed consent form (ICF), patients answered a questionnaire to assess their symptoms and collect other sociodemographic data. At their 4th treatment session, patients and their respective therapists were given an envelope containing the four self-report instruments. They were instructed to complete the instruments at the location of their choice and return them at the next session. Some cases were included in which the participants did not return the instruments at the 5th session but who, having spontaneously expressed their intention to bring them to the next session (the 6th session), did so. The remaining cases were excluded and treated as losses from the sample.

## Data analysis procedure

Data analysis was performed using SPSS 18.0 statistical software. Descriptive statistics were used to characterize the sample. Spearman correlations were used to investigate associations between variables. Hierarchical multiple linear regression was also used to obtain more specific information about relationships between the variables under study and the GSI, consisting of blocks. The choice of which variables to include in the model was not solely dependent on statistical associations in the bivariate analysis, but also on theoretical knowledge about the social and/or biological determinants of the events of interest. For this study, three blocks were created: in block I, variables such as sex and age, as well as parental attachment were included. In the next block (block II), traumatic events that could be influenced by variables in the upper block were included. In block III, defensive style variables were included, which can also be influenced by sociodemographic variables, parental bonding, and type of trauma. All variables that were included in the initial block were retained in the model until the end of inclusion of all blocks, regardless of statistical significance. The quality of fit of the model was ascertained according to the normality of the residuals and tests of homoscedasticity. In addition, multicollinearity between covariates was also tested. The level of significance (p) adopted in all analyses was 0.05.

## Ethical procedures

The investigation was conducted in compliance with ethical procedures established for research with human beings by the National Health Council (Resolution 196/96). The larger project was approved by CEP/UNISINOS; nº 14/184. All subjects involved in the research signed informed consent forms.

## Results

### Characterization of the sample

The sample consisted of 197 adult patients in Analytical Orientation Psychotherapy, 136 women and 61 men, aged between 18 and 67 years (mean [M] = 32; SD = 12.21). Most individuals who replied to the marital status question were married (24.9%) and a majority had higher education (74.0%), as shown in Table 1.

**Table 1:** Distribution of relative and absolute frequency of patients according to sociodemographic characteristics (n = 197)

	n	%
Sex		
Female	136	69.0
Male	61	31.0
Age		
Up to 30 years	14	55.1
31 to 50 years	63	33.4
Above 50 years	22	11.6
Missing	8	-
Education		
Elementary school	2	1.1
High school, complete	31	16.4
High school, incomplete	12	6.3
Higher education	140	74.0
Technical course	4	2.1
Missing	8	-
Marital status		
Single	17	21.8
Married	49	62.8
Widowed	9	11.5
Divorced	2	1.3
Stable relationship	1	2.6
Missing	119	-

### Descriptive analysis for the variables in study

In this study, descriptive analyses were performed of the results of the following instruments: CTQ, PBI, DSQ-40, and BSI, as shown in Table 2.

**Table 2:** Descriptive statistics of mean scores for the total scales and subscales of the instruments of trauma in childhood (CTQ), parental bonding (PBI), defensive styles (DSQ-40), and symptomatology (BSI)

	Minimum	Maximum	Mean	SD
CTQ				
Emotional abuse	1.00	4.60	2.11	1.00
Physical abuse	1.00	4.40	1.60	0.70
Sexual abuse	1.00	5.00	1.41	0.89
Emotional neglect	1.00	4.80	2.17	0.99
Physical neglect	1.00	3.60	1.42	0.58
CTQ total	1.00	4.36	1.75	0.65
PBI				
Care, mother	0.00	36.00	22.74	9.30
Control, mother	0.00	34.00	15.70	8.24
Care, father	0.00	36.00	19.90	9.60
Control, father	0.00	39.00	14.06	8.09
DSQ-40				
Mature	1.00	7.80	4.91	1.23
Neurotic	1.13	7.50	4.24	1.27
Immature	1.18	7.41	3.65	1.13
BSI				
Anxiety	0.00	4.00	1.24	0.87
Somatization	0.00	3.71	0.78	0.83
Psychoticism	0.00	3.80	0.98	0.84
Paranoid ideation	0.00	3.80	1.20	0.95
Obsession-compulsion	0.00	4.00	1.47	0.93
Hostility	0.00	3.80	0.99	0.75
Phobic anxiety	0.00	4.00	0.67	0.82
Depression	0.00	4.00	1.40	0.98
Interpersonal sensibility	0.00	4.00	1.29	0.99
GSI	0.02	3.60	1.12	0.72

BSI = Brief Symptom Inventory; CTQ = Childhood Trauma Questionnaire; DSQ-40 = Defensive Style Questionnaire; GSI = General Severity Index; PBI = Parental Bonding Inventory; SD = standard deviation.

The most frequent traumatic events in the sample, as assessed by the CTQ, were emotional neglect (M = 2.17; SD = 0.99) and emotional abuse (M = 2.11; SD = 1.00), followed by physical abuse (M = 1.60; SD = 0.70), physical neglect (M = 1.42; SD = 0.58), and sexual abuse (M = 1.41; SD = 0.90). Mean total trauma was 1.75 (SD = 0.65). It is important to note that only 5% of the patients reported never having had an adverse experience in childhood (a total CTQ score of 1). It was found that 84% of individuals reported abuse and emotional neglect, 53.3% reported physical neglect, 69% reported physical abuse, and 29% reported sexual abuse.

Regarding parental bonds, considering the cutoff points for the PBI dimensions, patients reported low maternal care (M = 22.74; SD = 9.30) and high maternal control (M = 15.70; SD = 8.24), as well as low paternal care (M = 19.90; SD = 9.60) and high paternal control (M = 14.06; SD = 8.09). The two PBI dimensions, care and control, identified the parental styles optimal parenting, affectionate constraint, affectionless control, and neglectful parenting. The most prevalent care style was control without affection for both mother and father. These results are shown in Table 3.

**Table 3:** Relative and absolute frequencies of parenting styles (PBI; n = 197)

	n	%
Parental bonding, mother		
Optimal parenting	43	22.6
Affectionate constraint	34	17.9
Affectionless control	75	39.5
Neglectful parenting	38	20
Missing	7	-
Parental bonding, father		
Optimal parenting	35	20
Affectionate constraint	34	19.4
Affectionless control	62	35.4
Neglectful parenting	38	20
Missing	15	-

PBI = Parental Bonding Inventory.

Regarding defenses, the mature defense style was the most prevalent in the sample (M = 4.91; SD = 1.23), followed by neurotic defenses (M = 4.24; SD = 1.27) and immature defenses (M = 3.65; SD = 1.13). Anticipation (M = 5.81; SD = 1.96) and humor (M = 5.29; SD = 2.30) were the most cited defenses from the mature defenses group. The most prevalent neurotic defense was pseudo-altruism (M = 5.00; SD = 1.77), followed by reaction formation (M = 4.77; SD = 1.94), and undoing (M = 3.93; SD = 2.05) and, finally, the most reported immature style defenses were rationalization (M = 5.03; SD = 1.88), somatization (M = 4.83; SD = 2.28), acting out (M = 4.25; SD = 2.15), and isolation (M = 4.21; SD = 2.21).

Considering the general BSI severity index and subscales, it can be observed that the most intense symptoms were obsession-compulsion (M = 1.47; SD = 0.93), depression (M = 1.40; SD = 0.98), interpersonal sensitivity (M = 1.29; SD = 0.99), anxiety (M = 1.24; SD = 0.87), and paranoid ideation (M = 1.20; SD = 0.95). The GSI averaged 1.12 (SD = 0.72).^7^

### Study of correlations between variables

Possible associations between childhood trauma, parental bonding, defensive styles, as well as the current symptoms of patients, were tested using Spearman’s correlation coefficients.

#### Childhood trauma and symptomatology

This analysis was performed previously in an earlier study with the same sample of patients by Waikamp & Serralta.^7^ It was observed that total trauma (total CTQ) presented positive and significant correlations with all dimensions of symptoms (BSI), as well as with the GSI (r = 0.37; p = 0.001). Considering each type of trauma, the strongest correlations found were: emotional abuse with paranoid ideation (r = 0.39; p = 0.001), with psychoticism (r = 0.32; p = 0.001), with interpersonal sensitivity (r = 0.34; p = 0.001), and with depression (r = 0, 33; p = 0.001); physical abuse with paranoid ideation (r = 0.21; p = 0.005) and with interpersonal sensitivity (r = 0.20; p = 0.006); sexual abuse with paranoid ideation (r = 0.20; p = 0.007) and with psychoticism (r = 0.18; p = 0.014); emotional neglect with paranoid ideation (r = 0.33; p = 0.001), with depression (r = 0.32; p = 0.001), with somatization (r = 0.28; p = 0.001) and with interpersonal sensitivity (r = 0.28; p = 0.001); and physical neglect with phobic anxiety (r = 0.28; p = 0.001) and with somatization (r = 0.28; p = 0.001).

#### Parental bonding and symptomatology

As shown in Table 4, it can be observed that maternal care was negatively correlated with paranoid ideation (p = 0.011), with obsession-compulsion (p = 0.035), and with somatization (p = 0.049). The dimension maternal control had positive and significant associations with all symptoms, especially with interpersonal sensitivity (p = 0.001), anxiety (p = 0.001), obsession-compulsion (p = 0.001), paranoid ideation (p = 0.001), and psychoticism (p = 0.023), and also with the GSI (p = 0.001).

**Table 4:** Correlations between parental bonding and symptomatology

Parental bonding (PBI)	Symptomatology (BSI)									

	ANX	SOM	PSY	PAR	OC	HOS	PHOA	DEP	IS	GSI
Care, mother	-0.07	-0.14*	-0.05	-0.18*	-0.15*	-0.10	-0.12	-0.12	-0.09	-0.14
Control, mother	0.26**	0.18*	0.16*	0.23**	0.25**	0.21**	0.17*	0.17*	0.27**	0.26**
Care, father	-0.15*	-0.14*	-0.15*	-0.11	-0.19*	-0.18*	-0.17**	-0.21**	-0.17*	-0.20**
Control, father	0.14	0.17*	0.16*	0.20**	0.22**	0.21**	0.05	0.17*	0.18*	0.20**

ANX = anxiety; BSI = Brief Symptom Inventory; DEP = depression; GSI = General Severity Index; HOS = hostility; IS = interpersonal sensitivity; OC = obsession-compulsion; PAR = paranoid ideation; PBI = Parental Bonding Inventory; PHOA = phobic anxiety; PSY = psychoticism; SOM = somatization.

** = < 0.01.

* = < 0.05.

On the father’s scale, the dimension care had negative correlations with several symptoms ranging from depression (p = 0.001) to somatization (p = 0.059) and with the GSI (p = 0.001). Paternal control, as well as maternal control, showed positive and significant correlations with a range of symptoms, especially obsession-compulsion (p = 0.001), hostility (p = 0.001), and paranoid ideation (p = 0.001), and the GSI (p = 0.001).

#### Defensive styles and symptoms

Considering the three defenses factors, these showed significant associations with most of the symptoms and the GSI (p = 0.001), (p = 0.001), and (p = 0.001), as shown in Table 5. In the mature defense style, negative correlations were observed with all symptoms, especially with depression (p = 0.001) and interpersonal sensitivity (p = 0.001). Neurotic defenses were also associated with a large proportion of the symptoms, ranging from psychoticism (p = 0.001), paranoia (p = 0.001) and hostility (p = 0.039). It was notable that the immature defense style had strong associations with all the BSI symptoms and the GSI (p = 0.001), highlighting the intense relationship with depression (p = 0.001), interpersonal sensitivity (p = 0.001), psychoticism (p = 0.001), paranoia (p = 0.001), obsession-compulsion (p = 0.001), and anxiety (p = 0.001).

**Table 5:** Correlation between defensive styles and symptomatology

Defensive styles (DSQ-40)	Symptomatology (BSI)									

	ANX	SOM	PSY	PAR	OC	HOS	PHOA	DEP	IS	GSI
Mature	-0.32**	-0.17*	-0.32**	-0.27**	- 0.33**	-0.32**	-0.28**	-0.44**	-0.39**	-0.39**
Neurotic	0.30**	0.22**	0.31**	0.31**	0.29**	0.15*	0.13	0.25**	0.31**	0.32**
Immature	0.53**	0.43**	0.60**	0.60**	0.58**	0.51**	0.35**	0.61**	0.61**	0.65**

ANX = anxiety; BSI = Brief Symptom Inventory; DEP = depression; DSQ-40 = Defensive Style Questionnaire; GSI = General Severity Index; HOS = hostility; IS = interpersonal sensitivity; OC = obsession-compulsion; PAR = paranoid ideation; PHOA = phobic anxiety; PSY = psychoticism; SOM = somatization.

** = < 0.01.

* = < 0.05.

#### Hierarchical multiple linear regression

As shown in Table 6, when referring to parental bonding, the subset that indicated maternal and paternal affectionless control had higher symptoms scores than those who reported optimal parenting by their fathers and mothers. As for the traumas reported, abuse and emotional neglect were the adverse experiences that most explained the symptoms observed in the patients. However, when all variables are included in the model, the mature and immature defense styles were sufficient to explain more than 50% (r^2^ = 0.575) of the variance in general psychopathology severity, reinforcing the hypothesis that defenses, except for neurotic defenses, explain the level of perceived psychological suffering caused by these patients’ current symptoms.

**Table 6:** Hierarchical multiple linear regression (n = 197)

	Multivariate hierarchical model					
	
	B	Beta	Sig	r^**2**^	Δr^**2**^	p-value
Block I - Sociodemographic variables and parental bonding				-	-	-
Sex	-0.116	-0.074	0.343			
Age	-0.011	-0.177	0.023			
Affectionate constraint, mother	0.213	0.116	0.209			
Affectionless control, mother	0.282	0.187	0.064			
Neglectful parenting, mother	-0.147	-0.079	0.418			
Optimal parenting, mother	**ref.**	-	-			
Affectionate constraint, father	0.172	0.095	0.324			
Affectionless control, father	0.382	0.247	0.019			
Neglectful parenting, father	0.300	0.181	0.086			
Optimal parenting, father	**ref.**	-	-			
Block II - Types of trauma				0.207	0.072	0.004
Emotional abuse	0.139	0.189	0.076			
Physical abuse	-0.020	-0.018	0.841			
Emotional neglect	0.208	0.273	0.038			
Physical neglect	-0.004	-0.003	0.974			
Block III - Defensive styles				0.570	0.363	0.000
Mature	-0.184	-0.312	0.000			
Neurotic	0.038	0.064	0.353			
Immature	0.374	0.556	0.000			

B = non-standardized coefficient; Beta = standardized coefficient; Sig = significance.

## Discussion

This research sought to examine the association between childhood trauma, parental bonding, defensive styles, and psychiatric symptoms in adult patients seen at an analytical psychotherapy clinic. The results obtained suggest that there are associations between these variables, reinforcing the clinical character of the processes that involve early traumas and their long-term repercussions.

The correlation between childhood trauma and psychiatric symptoms has already been reported by Waikamp & Serralta.^7^ More than half of the participants reported having been victims of neglect and emotional abuse. According to Bins et al.,^2^ these types of adversity are among the most difficult experiences for victims to identify and have been increasingly associated with catastrophic consequences in adult life. This form of violence demonstrates to children that their caregivers do not consider them to be worthy of love, which makes them feel unwanted. In this sense, according to a meta-analysis conducted by Zatti et al.,^34^ discrete forms of childhood trauma, such as cases of emotional neglect and a broken home, can significantly contribute to suicide attempts in adulthood.

Obsessive-compulsive symptoms were most prevalent in the sample, followed by depressive symptoms. These symptoms are predominantly related to a neurotic personality structure or organization, confirming the profile of patients who sought care at the clinic where this study was conducted.^7,35^

The powerful associations between abuse and emotional neglect and more serious symptoms such as paranoid ideation, interpersonal sensitivity, depression and psychoticism were notable, corroborating findings in the literature on the association between early trauma and psychotic symptoms^6,15,36^ and also with personality disorders.^15,21^ According to Siegel & Kohut,^37^ when studying severe symptoms, the individual is exempt from internalized object relations and there is a predisposition to emergence of psychotic symptoms. These symptoms would then be the individual’s attempt to recover contact with the lost objects. The typical symptoms of patients with severe personality disorders result from activation of the insecure attachment system that is also derived from adverse childhood experiences.^38^

Anxiety in adulthood was also directly associated with occurrence of traumatic experiences in childhood, particularly abuse and emotional neglect.^39^

As expected, the most prevalent care style in the sample was paternal and maternal affectionless control, which is characterized by high parental control (overprotection and intrusion) and low care (indifference and rejection). This type of bond represented by the caregivers’ lack of care and overprotection, is reflected in development of an insecure attachment in the child, resulting in damage to psychic resources, predisposing to psychopathology.^40,41^

Obsession-compulsion, paranoia, hostility, psychoticism, and the GSI were related to the control dimension of both parents, suggesting flaws in primary object relations, making the individual vulnerable to psychological suffering.^14,42^

Maternal control was the care style that showed the strongest relationship with the number of symptoms and also with the BSI GSI, which is consistent with work by Winnicott^43^ that indicates that failures in the mother’s ability to identify and be attentive to her baby’s needs predispose to psychopathology throughout the individual’s development. Therefore, the mother’s function has a decisive importance in her child’s life. These findings are in line with Catalan et al.^15^ and Marshall et al.,^16^ who found that maternal affectionless control was positively related to the sample’s current psychotic symptoms, as well as associated with depressive symptoms in adult individuals. In addition, intense associations were found between maternal control and patients’ interpersonal sensitivity. Although we did not find studies reporting this association, we can understand that controlling mothers can interfere too much in their children’s autonomy, not allowing them to make their own choices, which can be reflected in identity weaknesses, making it difficult for them to form and maintain affective bonds.

Negative correlations between paternal and maternal care and patients’ current symptoms, especially depression and paranoia, and also with the GSI, indicate that a bond characterized by love and support with primary object figures acts as a protective factor and contributes to the notion of psychological well-being and to establishment of stable relationships in adult life. Thus, care figures as a fundamental element in the constitution of individuals’ mental health.^13,43^

The intense negative correlation between paternal care and depression is noteworthy, suggesting the importance of the father’s role in development of depressive symptoms. According to Lebovici,^44^ the harmful consequences of deprivation of the paternal relationship with the child vary according to the degree of privation. Lack of this bond can cause anguish and depression.

Winnicott^45^ highlights the importance of a healthy environment, safe attachment style, and the good performance of vital roles by parents and/or caregivers for the child’s satisfactory psychological development. Taken together, the results of the present study emphasize the essential role that the relationship between parents/caregivers and children plays in these individuals’ future development.^46^

The mature defense style predominated in the sample studied. This can be explained by a possible neurotic organization of the patients’ personality and more mature defense mechanisms predominate, such as anticipation, humor, and suppression.^47^ McWilliams^35^ explains that this preference occurs unconsciously and is the result of interaction between several factors such as constitutional temperament, the tensions that this individual suffered in early childhood, the defenses modeled by caregivers, and the consequences of using private defenses. According to Eizirik & Bassols,^48^ the set of defenses individuals use to deal with anxiety derived from internal conflicts makes a decisive contribution to the structure of their personalities.

When correlated with symptoms, the immature defense style was the factor that most correlated with all symptom varieties, including the GSI. Corroborating findings reported by Gabbard,^21^ when the individual uses immature defenses, such as dissociation, they experience an illusion of control in the face of a traumatic situation, putting the event into perspective. As expected, mature defenses showed a negative correlation with all varieties of symptoms, as well as with the GSI. This suggests that the more mature and adaptive the individuals’ defenses are, the less susceptible they will be to psychological suffering, corroborating the literature.

### Predictors of symptomatology

Individuals who experienced unstable relationships with their caregivers needed to defend themselves from painful affective states derived from this failure of emotional restraint.^49^ These feelings not contained in the dyad are intensified by the child’s emotional responses, reinforcing their destructive potential. These experiences end up being internalized, impacting healthy development, which may disintegrate the ego. Therefore, there is a need to exclude these emotional states, as a way of protecting the ego from these pathogenic emotions. Early adversities lead to a negative perception of oneself and the other, leaving the individual unable to cope adaptively with their conflicts. Thus, there is a predominance of immature defenses for dealing with the perceived suffering derived from these early experiences.

In contrast, when children establish safe and supportive bonds with their caregivers, the caregivers can assist in regulation and containment of negative feelings, minimizing their effects.^12^ With this internalized relationship, the feeling of security is maintained and can reemerge throughout the individual’s life, without the caregivers’ presence, favoring emotional regulation. These experiences are fundamental for development of an intact and integrated ego, allowing individuals to deal with their emotional experiences more adaptively, helping in development of more mature defenses. Individuals with safe bonding experiences can deal with distressing emotional memories without being overwhelmed by them and without needing to use defenses that can distort reality.

## Conclusion

This study offers additional support for understanding the relationships between childhood trauma, experiences of bonding with parents in childhood, and defensive styles and the symptomatology of adult patients in analytically oriented psychotherapy. This is a correlational, inferential, and explanatory study and uses a hierarchical model that was based not only on the data, but also on theoretical hypotheses with a psychodynamic basis. In general, the results obtained suggest that there are associations between the variables, reinforcing the clinical character of the processes that involve early traumas and their long-term repercussions. The results obtained are in accordance with these assumptions and with empirical data from international studies that have examined these associations. It is important to highlight that the present study was carried out with a naturalistic sample, which explains its heterogeneity in terms of symptoms. Another important limitation of the study is related to the data collection procedures of the larger project from which this study is derived, in that instruments were applied during the fifth session in an uncontrolled environment. Moreover, this study used self-report measures, which can contribute to response bias due to the participants’ mental health status.
